# Depressive symptoms and physical function among the elderly in nursing homes during the COVID-19 pandemic in China: A cross-sectional study

**DOI:** 10.1097/MD.0000000000031929

**Published:** 2022-11-25

**Authors:** Lunan Gao, Jinhong Yang, Jiang Liu, Tingting Xin, Yuxiu Liu

**Affiliations:** a School of Nursing, Weifang Medical University, Weifang, China; b Department of oncology, Weifang People’s Hospital, Weifang, China.

**Keywords:** depression, nursing homes, risk factors, the elderly

## Abstract

The coronavirus disease 2019 (COVID-19) pandemic has placed a heavy burden on global healthcare. Depressive symptoms and physical function impairment are 2 common health problems among the elderly, but the association between depressive symptoms and physical function in nursing homes have not been extensively investigated during the COVID-19 pandemic. The purpose of this study was to investigate the current status of depressive symptoms and physical function and analyze the prevalence and related factors of depression among elderly people in nursing homes during the COVID-19 pandemic in China. A cross-sectional study was conducted. 381 elderly people were included in 4 nursing homes who were 60 to 100 years old with more than 3 months’ residential in Weifang City, Shandong Province using convenience cluster sampling. The Patient Health Questionnaire (PHQ-9) was performed to evaluate geriatric depression, the Barthel Index (BI) was administered to assess the activities of daily living, and a self-designed demographic data questionnaire was used to collect the demographic data. Multiple logistic regression analysis was conducted. 103 (27.0%) old residents reported depression according to PHQ-9. 279 (73.2%) old residents reported impaired self-care ability according to BI. The mean score of PHQ-9 and BI in the elderly was 3.56 ± 3.76 and 5.76 ± 7.05. The total PHQ-9 score of the elderly in nursing homes was positively correlated with the total activities of daily living score (*R* = 0.503, *P* < .01). Regression analysis showed that gender, self-care ability, more chronic diseases and medicines, especially Alzheimer’s disease and cataract were risk factors for depression among elderly people in nursing homes (*P* < .05). Our study showed 27.0% depression rate among old residents in nursing homes in China in the context of the COVID-19 pandemic. Depression is relatively prevalent among the elderly in China, and we should pay attention to those with poor self-care ability and more chronic diseases and medicines.

## 1. Introduction

Depression remains a great challenge to clinicians and imposes the risk of mortality and morbidity among the elderly.^[1–3]^ It is reported that depression was also one of the most common problems in the elderly in nursing homes besides chronic illness, and should be diagnosed and differentiated from other psychological illnesses.^[4]^ Nevertheless, geriatric depression is largely unexplored in nursing homes, partly due to their relatively occult and lack of specific performance.^[2,5]^ The prevalence of depression varies enormously depending on a lot of factors, especially where the elderly come from, for example, in homes, communities, or nursing homes.^[6]^ It was noteworthy that the severity of depression among the elderly in the nursing homes was more significant as compared to that of the community and homes.^[7–9]^ A meta-analysis indicated that the detection rate of depressive symptoms in nursing homes was 37.5%.^[10]^ Recent studies identified the prevalence of depression in homes, community, and nursing homes elderly people was 46.4%, 17.5%, and 57.1%, respectively in China.^[11–13]^ Currently, no consensus exists regarding the prevalence and risk factors for the elderly with depression. According to the reports above, there is a higher depression rate in nursing homes than compared in homes and communities, but there are few relevant reports, and more studies are needed to investigate the depression rate in nursing homes in China. In the present article, we assessed the prevalence and related factors of depression among elderly people in nursing homes in China.

Depression is generally associated with both internal and external factors including age, gender, and impaired cognitive and physical function.^[14–16]^ Previous studies found that elderly women are more at risk of depression,^[17,18]^ but in Patra’s and Tsai’s^[19,20]^ studies, gender was not a risk factor for depression among elderly people. It is necessary to further explore the risk factors of depression in the elderly in nursing homes, especially in the Chinese population where there is a lack of related reports. Dementia residents with high depression scores were more possible suffering behavioral dysfunctions and poorer physical health than the normal elderly,^[21]^ but most studies focused on the cognitive function of dementia patients to identify the correlation between depression and poor health symptoms not explore the specific cognitive disease, for instance, Alzheimer’s disease (AD) or Parkinson’s disease (PD). Other studies have reported the correlation between depression and poor health symptoms, including pain, sleep disturbances, and dyspnea.^[22–25]^ However, other chronic somatic diseases correlated with depression has been poorly researched in nursing homes, and more related researches are needed to better understand them.

The incubation period of the novel coronavirus disease 2019 (COVID-19) is 1 to 14 days, and people are generally susceptible. The outbreak occurred during the period of the Spring Festival (the Spring Festival is one of the most important festivals in China), with high personnel mobility, which has brought challenges to the prevention and control work. The elderly appeared strong susceptibility, most of them were serious, and symptoms not typical, so the elderly were regarded as the focus group by China State Council^[26]^ during the COVID-19 outbreak. The aging of the population poses a challenge that has never been faced before in China. According to the epidemiological study of 99 COVID-19 patients at an average age of 55.5 years, and patients aged 60 and above accounted for 37%.^[27]^ The elderly was under great psychological burden and mental and psychological pressure, and it was easy to have depression. As a protective measures, the Chinese government advocates home quarantine in the whole society, and anyone should wear masks when they leave homes and should not gathered together, in order to interrupt the transmission chain. According to previous studies reported that these measures lead to sadness and loneliness among older adults.^[28]^ However, the prevalence of depression and how it distributes among the elderly in nursing homes are not know. Specially, this study analyzed the complex relationships among depression, physical function, and chronic diseases in nursing homes based on Shandong province. Shandong is different from some of the other regions – such as Beijing and Shanghai – because the province has a higher proportion of aging population. Shandong is a major agricultural province, as the hometown of Confucius and Mencius, the birthplace of Confucian culture, has the largest total aged population and the second in terms of deep aging in China.^[29]^ With the influence of Chinese traditional filial piety culture and Confucian culture, most of the elderly are keen to live in the community or their own homes. Nursing homes are always the final choice of professional care for the elderly with severe functional or other physical limitations. The overwhelming evidence showed that there was a high rate of depression among the elderly in Chinese studies,^[9,14]^ and compared with other European countries, the number and severity of emotional diseases of the elderly in Chinese nursing homes had increased significantly.^[1,2]^

Therefore, this study investigated the psychological and physiological status of the elderly during the COVID-19 epidemic, in order to provide a basis for the formulation of psychological crisis intervention program for the elderly in emergencies.

## 2. Material and methods

### 2.1. Participants

Convenience cluster sampling was used. According to the standard put forward by Kendall (1975),^[30]^ the sample size was at least 5 to 10 times the number of items. Combined with the number of items in the questionnaire and the specific situation of the elderly in the nursing home, a total of 400 questionnaires were distributed. Questionnaires were distributed and collected on the spot in nursing homes. Three well-trained postgraduate nursing students then distributed questionnaires to the elderly who fulfilled the inclusion criteria. During the COVID-19 pandemic – the specified period of research, we invited to 400 eligible old adults, to fill out a survey questionnaire. Although illustrated exhaustively, nineteen of them refused to join the research because they were tired or uninterested, and only 381 old adults completed our questionnaire. This study was conducted among the elderly residents aged 60 years and above with 3 months or more living residence, who consent to participate in the study and came from 4 nursing homes in Weifang City, Shandong province from August to October 2021. The elderly who had serious diseases or cannot communicate clearly, such as deafness were excluded from this study. This study was conducted after Medical Ethics Committee of Weifang Medical University approval (approval number: 2019SL076). All participants were informed of the study content, and oral informed consent was obtained from themselves or their guardians.

### 2.2. Questionnaire

#### 2.2.1. *Resident-level factors*.

The data of resident-level factors were collected using the self-design assessment questionnaire, including age, gender, marital status, educational level, residence status, main income source, the number of children, the number of chronic diseases and medicines, and so on.

Chronic diseases were classified as nervous system including AD, PD, and stroke, circulatory system (hypertensive, myocardial infarction or interventional surgery history, heart failure, and pulmonary heart disease), respiratory system (asthma and chronic bronchitis), digestive system (liver cirrhosis and peptic ulcer), locomotor system-bone and joint, urinary system (cervical spondylitis, history of the lumbar spine, and rheumatoid arthritis), endocrine system (diabetes mellitus and thyroid disease), and other past and present medical history (cancer, cataract, infectious history, and history of intubation), including other common diseases.

#### 2.2.2. *Patient health questionnaire*.

The Patient Health Questionnaire (PHQ-9) was employed to measure depression.^[31]^ Cronbach’s alpha value was 0.886 in this study. The critical score was 5, 10, 15, and 20. No depression has a depression severity score between 0-4, mild depression between 5 and 9, moderate depression between 10 and 14, and moderately and severe depression is 15–19 and 20–27 respectively. A score of 5 or higher was regarded as depression.

#### 2.2.3. *Barthel Index*.

The activities of daily living (ADL) was administered to assess the elderly activity of daily living function by modified Barthel Index (BI).^[32]^ The modified BI is a 7-item scale except for unsuitable 3 items: bowels, bladder, stairs. Elderly people are advised to use the elevator instead of the stairs in nursing homes in China, we deleted item 10 walk up and down stairs in the original scale. The higher the score is, the worse the activity of daily living function is. Cronbach’s α coefficient of the scale in this study was 0.966.

### 2.3. Statistical analysis

Excel 2019 was used to establish the database and Statistical Package for Social Science version 21.0 for Windows (IBM Corp, Armonk, NY, USA) software was used for the elderly data analysis. Descriptive statistics were used to identify the characteristics of the elderly. Categorical variables were described by frequencies (percentages) and continuous variables were described by means (standard deviations). The differences among categorical variables were analyzed by chi-square tests. Pearson correlation coefficient was used to express the correlation between depression and ADL. Multiple logistic regression was employed to validate the correlation between geriatric depression and related factors in nursing homes. Odds ratio (OR) was used to present risk factors, with 95% confidence interval (95% CI). All tests were 2-tailed and statistically significant with *P* < .05.

## 3. Results

### 3.1. Baseline characteristics of the elderly

Of the 400 eligible elderly people who joined the investigation, a total of 19 questionnaires were excluded from the analysis because of incompleteness. The response rate was 95.3%, of which 381 were valid questionnaires. The average age of all the elderly was 82.25 ± 7.92 with a minimum of 60 and a maximum of 100 years. There were 312 (81.9%) old people who had chronic diseases, about 54.1% had 1 to 2 chronic diseases, and 20.1% had 3 to 4 diseases, 5.3% had 5 to 6 diseases. Table 1 shows the demographic characteristics of the elderly.

**Table 1:** General information of the elderly in nursing homes [n (%)].

Variables	No depression	Depression	*χ* ^*2*^	*P*
Age (yr)				
60–69	36 (87.8)	5 (12.2)	4.775	.189
70–79	70 (89.7)	8 (10.3)		
80–89	177 (89.8)	20 (10.2)		
90–100	52 (80.0)	13 (20.0)		
Gender				
Male	142 (84.0)	27 (16.0)	4.358	.037*
Female	193 (91.0)	19 (9.0)		
Marriage				
Single†	230 (71.9)	90 (28.1)	0.585	.444
Married	105 (86.1)	17 (13.9)		
Education background				
Undergraduate	31 (83.8)	6 (16.2)	6.696	.153
College	36 (100.0)	0 (0.0)		
High school	51 (86.4)	8 (13.6)		
Junior high school	64 (84.2)	12 (15.8)		
Primary school/no normal schooling	153 (88.4)	20 (11.6)		
Religious beliefs				
No	303 (88.3)	40 (11.7)	0.549	.459
Yes	32 (84.2)	6 (15.8)		
Previous employer				
Civil servants	153 (88.4)	20 (11.6)	1.304	.935
Soldier	15 (88.2)	2 (13.3)		
Professional and technical personnel	66 (88.0)	9 (12.0)		
Self-run, private enterprise	22 (88.0)	3 (12.0)		
Farming	73 (88.0)	10 (12.0)		
Other	6 (75.0)	2 (25.0)		
The main source of income				
Pension	252 (88.7)	32 (11.3)	2.629	.296
Children subsidies	74 (84.1)	14 (15.9)		
Other	9 (100.0)	0 (0.0)		
The way of payment for treatment cost				
Basic medical insurance system for urban workers and residents	270 (87.7)	38 (12.3)	2.424	.298
New rural cooperative medical care	16 (100.0)	0 (0.0)		
Other	49 (86.0)	8 (14.0)		
The number of children				
0–1	39 (79.6)	10 (20.4)	11.302	.004*
2–4	269 (90.9)	27 (9.1)		
≥5	27 (75.0)	9 (25.0)		
Residence before admission to nursing home				
Living alone	181 (92.3)	15 (7.6)	8.096	.017*
Living with spouse	95 (84.8)	17 (15.2)		
Living with children	59 (80.8)	14 (19.2)		
Reasons for entering pension institutions				
No children or children unable to take care of due to family or work	249 (89.2)	30 (10.8)	3.455	.178
Ask for professional care	79 (83.2)	16 (16.8)		
Other	7 (100.0)	0 (0.0)		
Check-in nursing homes time (yr)				
0–1	74 (89.2)	9 (10.8)	0.184	.912
2–3	124 (87.9)	17 (12.1)		
≥4	137 (87.3)	20 (12.7)		
Chronic diseases				
0	64 (91.4)	6 (8.6)	19.431	<.001*
1–2	191 (92.7)	15 (7.3)		
3–4	62 (77.5)	18 (22.5)		
≥5	18 (72.0)	7 (28.0)		
Medicines				
0	165 (92.2)	14 (7.8)	14.141	.003*
1–2	79 (91.9)	7 (10.5)		
3–4	63 (78.8)	17 (21.3)		
≥5	28 (77.8)	8 (22.2)		

* *P* < .05.

† Single including unmarried, divorce or widowed.

### 3.2. Prevalence of depression

The mean score of PHQ-9 in the elderly was 3.56 ± 3.76. About 73.0% of the elderly in nursing homes had no depression, and 27.0% had depression. Among them, there were 57 (15.0%) elderly with mild depression (PHQ-9 score 5–9), 28 (7.3%) elderly with moderate depression (PHQ-9 score 10–14), and 9 (2.4%) with moderately severe (PHQ-9 score 15–19) and severe depression (PHQ-9 score 20–27), respectively.

### 3.3. Prevalence of ADL impairment

The mean score of ADL in the elderly was 5.76 ± 7.05, and the number of nursing home residents with mild self-care ability disorder accounted for 42.5% with 26.8% maximum strength and independence, and 16.5% were maximum disability and dependency. The incidence of ADL impairment was highest in eating (70.9%) while it was least in locomotion, further information is presented in Table 2.

**Table 2:** Impairment of activities of daily living for the elderly in nursing homes.

Items	Completely self-care		Moderately self-care		Mostly independence		Completely independence	
	Number	(%)	Number	(%)	Number	(%)	Number	(%)
Hygiene	255	66.9	28	7.3	39	10.2	59	15.5
Toilet	247	64.8	42	11.0	29	7.6	63	16.5
Eating	270	70.9	29	7.6	35	9.2	47	12.3
Transfer	230	60.4	74	19.4	37	9.7	40	10.5
Locomotion	139	36.5	142	37.3	31	8.1	69	18.1
Dressing	233	61.2	57	15.0	37	9.7	40	10.5
Shower	161	42.3	92	24.1	68	17.8	60	15.7

### 3.4. The correlation between depression and ADL

Table 3 shows the results of correlation analysis showed that the total PHQ-9 score of the elderly in nursing homes was positively correlated with the total ADL score (*R* = 0.503, *P* < .01), indicating that the more severely damaged the ADL, the higher the depression level of the elderly in nursing homes.

**Table 3:** Correlation analysis of The Patient Health Questionnaire and Activities of Daily Living among the elderly in nursing homes.

	Item 1	Item 2	Item 3	Item 4	Item 5	Item 6	Item 7	Item 8	Item 9	PHQ-9
Item 1	0.413*	0.386*	0.312*	0.270*	0.389*	0.262*	0.426*	0.260*	0.248*	0.457*
Item 2	0.393*	0.369*	0.263*	0.240*	0.318*	0.226*	0.395*	0.255*	0.203*	0.411*
Item 3	0.437*	0.405*	0.308*	0.264*	0.373*	0.271*	0.410*	0.316*	0.251*	0.465*
Item 4	0.442*	0.445*	0.319*	0.298*	0.392*	0.242*	0.431*	0.271*	0.226*	0.476*
Item 5	0.426*	0.413*	0.286*	0.317*	0.351*	0.242*	0.428*	0.269*	0.218*	0.458*
Item 6	0.476*	0.444*	0.345*	0.350*	0.354*	0.225*	0.463*	0.334*	0.209*	0.498*
Item 7	0.414*	0.407*	0.260*	0.271*	0.339*	0.247*	0.382*	0.265*	0.241*	0.435*
ADL	0.476*	0.456*	0.331*	0.311*	0.395*	0.264*	0.467*	0.302*	0.246*	0.503*

ADL = activities of daily living, PHQ-9 = The Patient Health Questionnaire.

**P <.01.

### 3.5. Factors associated with depression

The chi-square test showed that the difference in demographic variables, including gender, the number of children, residence before admission nursing home, chronic diseases and medicines were regarded as statistically significant (*P* < .05) between depression and non-depression elderly (Table 2). Table 4 shows the correlation between chronic diseases and depression in the elderly in nursing homes, including AD, BHP, and cataract. The above 7 variables (gender, the number of children, residence before admission to nursing home, medicines, AD, BHP, and cataract) were incorporated in the logistic test model. In multiple logistic regression, the ADL level (OR = 4.942, 95% CI [3.049, 8.008]), AD (OR = 2.479, 95% CI [1.119, 5.572]), male (OR = 3.234, 95% CI [1.096, 9.541]), cataract (OR = 14.739, 95% CI [3.006, 72.263]) were the risk factors for depression among the elderly living in nursing homes (Table 5).

**Table 4:** Correlation between chronic diseases and depression of the elderly in nursing homes.

System	No depression (%)	Depression (%)	*χ* ^*2*^	*P*
Nervous system				
AD	28 (75.7)	9 (24.3)	4.586	.032*
PD	55 (88.7)	7 (11.3)	1.428	.232
Sequelae of stroke	55 (88.7)	7 (11.3)	0.043	.836
Circulatory system				
Hypertension	187 (88.6)	24 (11.4)	0.218	.641
Myocardial infarction	56 (91.8)	5 (8.2)	1.028	.311
Cardiac failure	12 (85.7)	2 (14.3)	0.000	1.000
Pulmonary heart disease	2 (66.7)	1 (33.3)	0.257	.321
Respiratory system				
Asthma	15 (88.2)	2 (11.8)	0.000	1.000
Chronic bronchitis	20 (83.3)	4 (16.7)	0.152	.697
Digestive system				
Peptic ulcer	21 (84.0)	4 (16.0)	0.094	.760
Cirrhosis	4 (80.0)	1 (20.0)	1.000	.476
Motor system				
Cervical spondylosis	10 (90.9)	1 (9.1)	0.000	1.000
Lumbar spondylosis	27 (84.4)	5 (15.6)	0.130	.718
Rheumatoid arthritis	14 (93.3)	1 (6.7)	0.063	.801
Urinary system				
Urinary tract infections	12 (85.7)	2 (14.3)	0.000	1.000
Urinary calculi	1 (100.0)	0 (0)	1.000	.879
BPH	5 (55.6)	4 (44.4)	6.244	.012*
History of chronic kidney disease	2 (100.0)	0 (0.0)	1.000	.773
Endocrine system				
DM	88 (89.8)	10 (10.2)	0.434	.510
Thyroid disease	1 (100.0)	0 (0.0)	1.000	.879
Other				
Cancer	1 (50.0)	1 (50.0)	0.227	.227
Cataract	9 (52.9)	8 (42.1)	17.211	<.001
The history of intubation	5 (100.0)	0 (0.0)	1.000	.524

AD = Alzheimer’s disease, BPH = benign prostatic hyperplasia, DM = diabetes mellitus, PD = Parkinson’s disease.

* *P* < .05.

**Table 5:** Logistic regression analysis on the influencing factors of depressive symptoms among the elderly in nursing homes.

Variables	*β*	Wald *χ*^*2*^	OR	95% CI	*P*
ADL level	1.598	42.073	4.942	3.049–8.008	<.001
Gender	0.915	4.994	2.479	1.119–5.572	.025*
AD	1.174	0.552	3.234	1.096–9.541	.033*
Cataract	2.690	11.002	14.739	3.006–72.263	.001
Constant	-7.784	51.147	0.000	–	<.001

95% CI = 95% confidence interval, AD = Alzheimer’s disease, ADL = activities of daily living, OR = odds ratio.

* *P* < .05.

## 4. Discussion

This cross-sectional survey explored the prevalence and risk factors of geriatric depression in nursing homes in the context of the COVID-19 pandemic. Our findings showed that 27.0% of the elderly in nursing homes had depression symptoms. This result is consistent with other cross-sectional studies that reported depression rates of 18.5% to 32.8% in nursing homes for elderly people in different countries.^[1,3,33,34]^ This means that about 3 of ten elderly people have feelings of loneliness and sadness. The phenomenon could be attributed to the fact that the elderly admission into nursing homes requires facing an unfamiliar living environment and relationships while being limited by the physical disability,^[16,35]^ especially Chinese elderly people influenced by traditional Confucian culture^[36]^: “being attached to one’s native land builds up this homesick mode and make people generally unwilling to leave their hometown” – which has been referred to people be reluctant to move from one’s familiar and native district, and “rear sons for help in old age” – meanings old people had raised their children when their strong, also hope their children in turn, support them in old age. Additionally, this result can be explained that most nursing homes have forbidden the elderly to visit relatives and join social activities in China, so old people are completely isolated in nursing homes, and the frequency of communicated with others, especially their families were decreased. Due to the closed management and other reasons during the epidemic period of COVID-19, the elderly could not reunite with their families, which also increases their loneliness. Taken together, despite differences in sample and setting, the use of the different depression assessment tools in each study needs to be factored into consideration. Our study applied the PHQ-9, in which some items were involved in sleep quality and nutrition assessment. Therefore, these findings may be interpreted cautiously.

To our knowledge, this is the first cross-sectional research on depression status quo and related factors during COVID-19 in nursing homes in China. In this research, more risk factors such as more chronic diseases and elderly personal factors were revealed by performing chi-square test and multivariate analyses. The majority of this study concluded that gender, a higher score on the ADL, having more diseases, especially, AD, and cataract were more likely associated with depression in the elderly living in nursing homes.

The elderly gender is a predictor that impacts geriatric depression. In our study, a significant relationship between males and depression is reported. This finding is inconsistent with that of Carifio et al,^[37]^ who reported gender had no main significant effect on the level of depression in nursing homes by using Geriatric Depression Score. Conversely, approximately 19.0% of males showed depression in our sample, whereas only 9.8% of females have depression. Our result confirmed that males were more likely to get depression than females in nursing homes. This result may be explained by the feature that males in nursing homes always are widowers in this research, losing spouses’ care, may make them lonelier. Conceivably, having benign prostatic hyperplasia increases the frequency of urination, especially at night which may influence the quality of male residents’ sleep quality. Previous studies showed that there is a clear link between sleep quality and depression.^[38,39]^ Poor sleep quality is an important cause of depression, which will further aggravate the severity of depression.^[40]^ On the contrary, females are more likely to show higher social engagement, for example, more phone calls with their families than males, and higher ability daily levels in our study. Especially the closed management in the COVID-19 pandemic, this means fewer activities and opportunities for contact and communication with the outside world, making males prone to loneliness, and leading to depressive symptoms.^[16]^ Despite many studies on gender differences in depression, it has yet not been confirmed whether the incidence rate of geriatric depression is higher in elderly women than men. On the other hand, the variations in the sensitivity and specificity of various screening tools could result in a systematic difference in the prevalence rates of depression.

Our study showed that 73.2% of the nursing homes elderly also experienced ADL impairment, and 42.5% had a mild ADL impairment. The result indicates a positive association between a higher score of ADLs and geriatric depression. This means that elderly people who have a higher score of ADLs that less possibly care for themselves may act as a negative factor for depression. We found a significant association between physical function impairment and depression, which is consistent with that of Kaup et al^[41]^ and Djernes.^[6]^ This study concluded that physical function impairment elderly individuals were more likely to have depression than normal elderly people. This result may be explained that feeling disabled or dependent on someone else in daily activities such as walking, going toilet, and dressing may contribute to self-accusation and feelings of shame possibly further causing depression. This finding is similar to previous studies which showed impaired ADL in later life disrupted their sense of control over life and other psychological states resulting in depressive symptoms.^[42–45]^

This study identified that negative emotion was related to AD. Many studies that showed a clear correlation between depression and dementia.^[46,47]^ However, we further highlighted the risk of depression with AD other than dementia caused by PD or sequelae of stroke. The logistic regression analysis verified that the odds of the elderly with AD getting depression were 3.2 times higher than the elderly without AD. The causal relationship between dementia and depression remained unclear and possibly bidirectional, which needed more relative research to better understand them.^[34]^

Besides, we also confirmed that the odds of elderly with cataract getting depression were 14.7 times higher than those without cataract. A systematic review also reported that depression was associated with visual impairment in the elderly.^[48]^ This finding may be explained by the fact that eye disorders may contribute to the avoidance of social situations, resulting in social isolation especially in the COVID-19 pandemic, and then leading to depression.

This study reported that depression of the elderly in nursing homes was at a comparatively high level in China during the COVID-19 epidemic, and this level of depression was of concern because psychological problems may further lead to decreasing physical function and worsening of the disease. Ulbricht et al^[49]^ also reported an analysis of 783,829 old adults in the US, identified residents often suffered from combinations of depression and other unhealthy emotions at admission to a nursing home, and they also found that the receipt of psychological therapy or any psychiatric medication was lacking. In this context, the elderly in nursing homes exhibited a high need for standardized evaluation procedures and intervention with cost-effective strategies inside and outside of nursing homes. Therefore, timely and appropriate mental nursing and depression assessments should be provided by professional clinicians in the development of nursing homes’ geriatric care in the future.^[50]^ For example, increasing the number of new social activities as long as it’s safe and reducing physical pain may be effective interventions.^[46,51]^ Due to the outbreak of the epidemic, the elderly has a weak resistance. On the 1 hand, they are afraid of family worries, and on the other hand, they need support from relatives. In the face of setbacks and difficulties, cannot be the same as usual to their most trusted family talk, this will undoubtedly increase the psychological pressure burden of the elderly. Under the condition of high stress, if an individual lacks support and cannot respond to stress in a positive way, the risk of psychological damage can reach 43.3%, which is more than twice the risk of ordinary people.^[52]^ Therefore, it is suggested that nursing homes’ managers take the initiative to assume the roles of family members, listeners and supporters of the elderly, earnestly care about their mental and physical health. Especially in the case of lack of food supplies and limited logistics, giving full help and support to families of the elderly in difficulty and solving their worries is also the key to relieve their psychological pressure. Nevertheless, there is a dearth of evidence-based medical evidence about risk factors associated with depression in nursing home elderly people, and more scientific studies are urgent especially at the special period.

Some limitations in the current study should be noted. Firstly, the sample seems to represent part of nursing home elderly people concerning depression and physical function. Besides, the cross-sectional study could just reveal a section of the influencing factors associated with depression in nursing homes, suggesting that subsequent studies should carry out corresponding longitudinal studies on depression in nursing homes. Last, the elderly who participated in our study in nursing homes may be more frailty and feel lonelier than community or home dwelling older people. Thus, the generalizability of our results to other settings should be cautious. Despite the limitations, in this research, our results offered primary data that would perform to explore personal nursing intervention in the elderly in nursing homes in emergencies in China.

## 5. Conclusion

In this study, we found that the incidence rate of geriatric depression in nursing homes in China is high. Special attention should be given to the elderly with many chronic diseases, for example, AD and cataract. Our study also confirms that those who have different levels of ADLs or physical ability impairments and have more medicines appear to be easy to depression. The results of our study suggest timely and appropriate mental nursing and depression assessment are needed to implement by clinicians working in nursing homes in the development of nursing homes geriatric care in the context of the COVID-19 pandemic. To some extent, this study reflects the psychological status and self-care ability of the elderly in nursing homes during the epidemic period of COVID-19, and provides a reference for nursing home managers to implement targeted intervention measures for the elderly. More longitudinal studies are needed to explore related factors for depression in the elderly to administer future interventions to improve mental health in the elderly in nursing homes.

## Acknowledgments

We would like to gratefully acknowledge the older adults who participated in this study.

## Author contributions

**Conceptualization:** Lunan Gao, Jinhong Yang, Jiang Liu, Tingting Xin.

**Data curation:** Lunan Gao, Jinhong Yang.

**Formal analysis:** Lunan Gao, Jinhong Yang, Jiang Liu.

**Investigation:** Lunan Gao, Jiang Liu, Tingting Xin.

**Methodology:** Lunan Gao, Jiang Liu, Yuxiu Liu.

**Supervision:** Yuxiu Liu, Yuxiu Liu, Jinhong Yang.

**Writing – original draft:** Lunan Gao, Jinhong Yang, Jiang Liu, Tingting Xin, Yuxiu Liu.

**Writing – review & editing:** Lunan Gao, Jinhong Yang, Jiang Liu, Tingting Xin, Yuxiu Liu.
